# Supplementary material for: Feeling safe or falling through the cracks—Patients’ experiences of healthcare in cirrhosis illness: A qualitative study
Source: PLoS One. 2023 Apr 5;18(4):e0283611. doi: 10.1371/journal.pone.0283611 (PMC10075419; doi:10.1371/journal.pone.0283611)
Supplement: Attachment — Submitted filename: Response to Reviewers.docx [file pone.0283611.s001.docx]

**Response to reviewer on manuscript PONE-D-22-11597: Well received or falling through the cracks – patients’ health care experiences with liver cirrhosis illness: A qualitative study**

Dear Editor,

We thank the reviewer for comments in order to improve our manuscript, entitled “Well received or falling through the cracks – patients’ health care experiences with liver cirrhosis illness: A qualitative study” (PONE-D-22-11597). We have revised the manuscript, and our answers to the suggestions are listed below. According to the reviewer´s suggestion, we have reanalysed the data. To be concurrent with the findings in the reanalysis, the title has been changed to “Feeling safe of falling through the cracks – patients’ health care experiences with liver cirrhosis illness: A qualitative study”. Further, the COREQ checklist has been updated accordingly.

We hope you will find our manuscript satisfactory for publication in PLOSONE.

Best regards,

The Authors

| EDITOR |  |
| --- | --- |
| the discussion is repeating the results without presenting new information | Please see discussion below in answers to reviewer’s comments |
| the section applying person-centred care would benefit from a re-worked analysis | Please see discussion below in answers to reviewer’s comments |
| whether the data presents a large enough variation in patients’ health care experiences in relation to LC | Please see discussion below in answers to reviewer’s comments |
| Please ensure that your manuscript meets PLOS ONE's style requirements, including those for file naming. The PLOS ONE style templates can be found at  <https://journals.plos.org/plosone/s/file?id=wjVg/PLOSOne_formatting_sample_main_body.pdf> and  <https://journals.plos.org/plosone/s/file?id=ba62/PLOSOne_formatting_sample_title_authors_affiliations.pdf> | Thank you for your reminder; this has been taken into account. |
| We noted in your submission details that a portion of your manuscript may have been presented or published elsewhere. Please clarify whether this publication was peer-reviewed and formally published. If this work was previously peer-reviewed and published, in the cover letter please provide the reason that this work does not constitute dual publication and should be included in the current manuscript. | The present manuscript’s data have ***not*** been published or submitted elsewhere.  - We guarantee that reference 3 had a different aim (*how persons living with liver cirrhosis experience day-to-day life*), than the aim of the present manuscript (*patients’ health care experiences in relation to liver cirrhosis illness*). Hence, despite the joint interview data collection, reference 3 and the present manuscript used completely different data to answer their different aims.  - The same goes for reference 18, which is a study protocol for a survey regarding a randomised controlled study. A forthcoming study will use all other survey data *except* the one from the two open-ended questions in the current publication. Hence, we also guarantee that the survey data have ***not*** been published or submitted elsewhere. |
| Please note that in order to use the direct billing option the corresponding author must be affiliated with the chosen institute. Please either amend your manuscript to change the affiliation or corresponding author, or email us at [plosone@plos.org](mailto:plosone@plos.org) with a request to remove this option. | All authors are researchers at Uppsala University. Centre for Clinical Research in Region Dalarna, which is Maria Hjorth’s affiliation, belongs to the Department of Medical Sciences, Uppsala University. Hence, Maria Hjorth’s affiliation is with both Centre for Clinical Research in Region Dalarna AND Dept. of Medical Sciences, Uppsala University. |
| REVIEWER 1 | Thank you for your helpful suggestions to further improve our manuscript. |
| 1. Is the manuscript technically sound, and do the data support the conclusions?  The manuscript must describe a technically sound piece of scientific research with data that supports the conclusions. Experiments must have been conducted rigorously, with appropriate controls, replication, and sample sizes. The conclusions must be drawn appropriately based on the data presented. Reviewer #1: Yes | N/A |
| 2. Has the statistical analysis been performed appropriately and rigorously? Reviewer #1: N/A | N/A |
| 3. Have the authors made all data underlying the findings in their manuscript fully available?  The [PLOS Data policy](http://www.plosone.org/static/policies.action#sharing) requires authors to make all data underlying the findings described in their manuscript fully available without restriction, with rare exception (please refer to the Data Availability Statement in the manuscript PDF file). The data should be provided as part of the manuscript or its supporting information, or deposited to a public repository. For example, in addition to summary statistics, the data points behind means, medians and variance measures should be available. If there are restrictions on publicly sharing data—e.g. participant privacy or use of data from a third party—those must be specified. Reviewer #1: Yes | N/A |
| 4. Is the manuscript presented in an intelligible fashion and written in standard English?  PLOS ONE does not copyedit accepted manuscripts, so the language in submitted articles must be clear, correct, and unambiguous. Any typographical or grammatical errors should be corrected at revision, so please note any specific errors here. Reviewer #1: Yes | N/A |
| 5. Review Comments to the Author |  |
| 1. The study presents the results of original research. Yes, this is original research. | N/A |
| 2. Results reported have not been published elsewhere. Yes | N/A |
| 3. Experiments, statistics, and other analyses are performed to a high technical standard and are described in sufficient detail. High technical standard and in sufficient detail.  Perhaps clarify how many interviews and questionnaire in one sentence at the data collection and place the sentence on raw 151 as the first sentence at data analysis—In total…. | According to your suggestion, the first sentence in the data collection section now clarifies the number of interviews and questionnaires that were collected (page 7, row 149).  We have rephrased the following sentences in the data collection section: “The 18 interviews…” (page 7, rows 149-150);  “The two open-ended questions, answered by 86 participants…” (page 7, rows 157-158); Further, the total number of participants is stated in the first sentence in the analysis section (page 8, row 164). |
| Please clarify the 6 steps in the analysis to show that there was TA analysis, as it is presented now it is very similar to content analysis. For example, initial codes are produced from data in step 2 and data is coded into so many patterns as possible. Etc… | We have further clarified the six analysis steps according to our methodological reference Braun and Clarke (2006):  1. Familiarising with data  2. Assigning codes generously describing the patterns of data  3. Searching for themes  4. Reviewing themes  5. Defining and naming themes  6. Producing the report with extracts embedded in an analytic narrative, going beyond descriptions of data.  Please see page 8 rows 165-177.  To comply with the analytical method, we have clarified that we use “an interpretative descriptive design” in the study design section (page 5, 110) |
| The aim was to describe patients’ health care experiences in relation to liver cirrhosis illness. The title is; Well received or falling through the cracks – patients’ health care experiences with liver cirrhosis illness: A qualitative study | To be concurrent to the revised analysis, we have changed the title to: “Feeling safe of falling through the cracks – patients’ health care experiences with liver cirrhosis illness: A qualitative study”, please see page 1, row 2-4.  Also the aim of the study has been changed to: “To capture patients’ descriptions…” (page 2, row 32; page 5, row 106; page 14, row 339) |
| Data collection, more information is supplied in reference 3.- so this is a secondary analysis of that interview data? In reference 3 data was analysed with content analysis- was there enough data to make a secondary analysis, with another perspective and with some interpretation attached? Please, clarify. The interview questions in reference 3 have focus on life situation, how could data respond to health care experiences? | Thank you for encouraging us to clarify this issue throughout the text:  - In the sample and setting section, we have clarified that 18 out of 20 interviews *spontaneously* described experiences regarding healthcare (page 5, row 124).  - We have moved two sentences from strength and limitations to data collection, to be transparent and direct about the data collection procedure. The new sentences are: “The interviewer did not ask about healthcare experiences. Instead, informants spontaneously reported their healthcare experiences in parallel with answers to questions about experiences of day-to-day life in relation to LC illness. For these people, patient care was part of life and was described in broad variations” (page 7, rows 150-153).  - In the strength and limitation section, we have reformulated to: “Although the interview questions did not intend to explore patients’ healthcare experiences, data involved rich experiences to answer the current study aim. Also, during the interviews, the patients returned to their experiences of LC care repeatedly” (page 17, rows 401-404). |
| Trustworthiness is presented, according to reference 30 | N/A |
| The result is two themes, Being engaged, collaborating and feeling well-received and Feeling nervous, lost and receiving insufficient information. The result presentation is clearer if the sub-themes are left out- presenting only two themes are more story telling according to TA (thematic analysis). One reflection here, these two themes seem to be two sides of the same coin and they are just piled sub-themes labels- they are not presenting the meaning or the content of the theme (according to TA) | According to your suggestion, we have now reanalysed our data to improve the interpretation and the meaning of the story told by the data, according to Braun and Clarke (2006). Please see the result section page 9 row 206 to page 14, row 337; and Figure 1. Changes have also been made in the abstract, discussion and conclusion in order to clarify our new result (page 2, rows 38–41 and rows 43-46; page 14, rows 339–341; page 15, rows 343–346, rows 348–350, rows 354-362, rows 367-377; page 16 rows 380-382; page 18, rows 424–426, rows 430-435). We argue that the use of sub-themes helps the reader to understand the important message of the results. |
| I think that there is a need to re-work the analysis, the information in data seem to be about a struggle to be in dialogue (getting information, being involved, and perceived as a person) and there is uncertainty about the health care system (ignorance about the disease, no continuity and not being taken care of). Re-working the analysis should strengthen the result. | We agree with the first suggested theme ‘Struggle to be in a dialogue’. Data, however, do not give support to the second suggested theme ‘Uncertainty about the health care system’. Instead, we have chosen to reformulate the theme pursuant to the data. The second theme has now been labelled ‘Being helped or harmed’. Please see the result section, page 9 row 206 to page 14, row 337 and Figure 1. |
| This study is focusing on an important and interesting area of research, so if the analysis were analysed according to TA there would be interesting information. | Thank you. We have considered the comments regarding the analysis, and elaborated the 5^th^ and 6^th^ stage of the analysis according to Braun & Clarke (2006). |
| The discussion is repeating the result and confirmed by references used, nothing new presented, but this could be due to the “directed analysis”. The section applying person-centred care is interesting and would benefit of a re-worked analysis. | Thank you for raising this issue. Our intention of using Santanas’s framework for person-centred care was solely for the discussion of the results. In the introduction of Santana’s framework, we have now clarified our intention (see page 4, rows 81-83). We have reviewed the discussion section in order to shorten our own findings. Further, we have strived to articulate the news and importance of our findings (see page 15, rows 343–346, rows 348-350, rows 354-362 and rows 367-377). |
| Limitations are well presented but I am not sure that data presents a large variation of patients’ health care experiences in relation to LC. This section could be shortened- it is repeating some information and some information should be placed at data collection section etc. | The limitation section has been shortened and reviewed to avoid repetition. In the first sentence in the analysis section, we have clarified the amount of data that covers our thematic analysis (see page 8, rows 164–166). We have also moved some text from the limitation section to the data collection section, according to your suggestions (see page 7, rows 156-158). We have clarified new quality aspects of the data in the strength and limitation section (see page 17, rows 401–404). |
| 4. Conclusions are presented in an appropriate fashion and are supported by the data. Conclusions are presented in appropriate fashion, but it is a repeated result in short-short version. Even so, there are some highlights about uncertainty and so on, but this is not mentioned in the result…. | Since our experience is that many researchers search through voluminous articles and sometimes only scan the articles’ objectives, followed by the conclusion, before deciding to read further or quit, we’re of the opinion that a conclusion section should have a short repeat of the main results, followed by a conclusion twist. We hope you agree with this proposition.  Concerning the message of uncertainty, we hope you consider this to be more evident in the reworked result section (see page 11, row 241 and rows 250–254; page 13, rows 311–312). |
| 5. The article is presented in an intelligible fashion and is written in standard English. Yes, the article is presented in an intelligible fashion, and it is written in standard English | Thank you |
| The introduction is well-written and structured, leading me as a reader forward to the aim. | Thank you very much |
| 6. The research meets all applicable standards for the ethics of experimentation and research integrity. Yes, this study meets all the applicable standards for research integrity | N/A |
| 7. The article adheres to appropriate reporting guidelines and community standards for data availability. Yes, the article is following the reporting guidelines. | N/A |
| This could be an interesting paper, presenting important knowledge. | Thank you |
| This paper needs to be re-analysed then it would be suitable for publication. | We have made a major and robust re-analysis, and hope you’ll find it satisfactory. |
| Out of 37 references 10 are 10 years old or more, there are 23 references in the introduction and out of these 5 are 10 years old or more (mostly instruments references). | We have replaced one reference in the discussion section with reference 36, year 2017. The introduction references, older than 10 years, however, regard important concept definitions and a WHO position paper, which are hard to replace. The same applies to five methodological references that are older than 10 years, which we believe there are compelling reasons to use. |
