# Supplementary material for: Feeling safe or falling through the cracks—Patients’ experiences of healthcare in cirrhosis illness: A qualitative study
Source: PLoS One. 2023 Apr 5;18(4):e0283611. doi: 10.1371/journal.pone.0283611 (PMC10075419; doi:10.1371/journal.pone.0283611)
Supplement: Attachment — Submitted filename: Response to reviewers.docx [file pone.0283611.s002.docx]

**Response to reviewer on manuscript PONE-D-22-11597: Feeling safe or falling through the cracks – patients’ healthcare experiences with cirrhosis illness: A qualitative study**

Dear Editor,

We thank the reviewer for the second round of comments in order to improve our manuscript, entitled “Feeling safe or falling through the cracks – patients’ healthcare experiences with cirrhosis illness: A qualitative study” (PONE-D-22-11597). According to the reviewers suggestions, we have thoroughly revised the introduction and discussion section and made supplementary changes in the manuscript as requested. Also, we have made a rewording of the title to “Feeling safe or falling through the cracks – patients’ healthcare experiences in cirrhosis illness: A qualitative study”. Each reviewer’s comment and our responses are listed below. Further, all changes are marked with track changes in the manuscript, which also includes some words that were not commented upon by the reviewers. Accordingly, the COREQ checklist has been updated.

We hope the improvements of the manuscript are satisfactory.

Best regards,

The Authors

| EDITOR |  |
| --- | --- |
| Thank you for submitting your manuscript to PLOS ONE. After careful consideration, we feel that it has merit but does not fully meet PLOS ONE’s publication criteria as it currently stands. Therefore, we invite you to submit a revised version of the manuscript that addresses the points raised during the review process. | Thank you for the opportunity to clarify and address the outstanding issues in our manuscript. We have thoroughly responded to each of the reviewers’ suggestions below. We hope that the improvements meet your expectations for publication in PLOS ONE. |
| The reviewers have raised a number of concerns that need attention. They request additional information on methodological aspects of the study (such as how the participants were selected and specifics of the theoretical approach taken with respect to the qualitative analyses), clarification in the results section (including which data are from which set of participants), and request revisions to the introduction and discussion sections. Could you please revise the manuscript to carefully address the concerns raised? | Please see our response to each of the reviewer’s comments below. |
| **REVIEWER 1** |  |
| If the authors have adequately addressed your comments raised in a previous round of review and you feel that this manuscript is now acceptable for publication, you may indicate that here to bypass the “Comments to the Author” section, enter your conflict of interest statement in the “Confidential to Editor” section, and submit your "Accept" recommendation.  Reviewer #1: All comments have been addressed | Thank you |
| Is the manuscript technically sound, and do the data support the conclusions? Reviewer #1: (No Response) | N/A |
| Has the statistical analysis been performed appropriately and rigorously?  Reviewer #1: (No Response) | N/A |
| Have the authors made all data underlying the findings in their manuscript fully available?  Reviewer #1: (No Response) | N/A |
| Is the manuscript presented in an intelligible fashion and written in standard English?  Reviewer #1: (No Response) | N/A |
| Thank you for your efforts in amending and clarifying the manuscript. All comments have been taken into consideration. I am looking forward to see it published | Thank you |
| **REVIEWER 2** | Thank you for your suggestions on further improving our manuscript. We appreciate your clearly stated questions on the topics below. |
| Is the manuscript technically sound, and do the data support the conclusions? Reviewer #2: Yes | N/A |
| Has the statistical analysis been performed appropriately and rigorously?  Reviewer #2: N/A | N/A |
| Have the authors made all data underlying the findings in their manuscript fully available?  Reviewer #2: No | N/A since there are ethical considerations limiting our possibility to share data, which has been described previously. |
| Is the manuscript presented in an intelligible fashion and written in standard English?  Reviewer #2: Yes | N/A |
| The authors tackle an important area of study in cirrhosis care. In order for the healthcare system to provide more patient-centered care, studies such as this one are needed to focus our care improvement problem. While I was not a primary reviewer, I do see that the authors have significantly edited the paper. Despite this, I would recommend further revisions based on my comments below. | Thank you, we have strived to improve our manuscript based on the questions raised. |
| *General comments:* |  |
| “Being helped or harmed” – not sure this theme name carries a meaning that is obvious; perhaps something about Benefits and Burden of care since the authors include subthemes of burden of care. | According to the previous PlosOne reviewer’s comment, we changed the theme to ‘Being helped or harmed’ after consulting data. However, we have considered your suggestion, but conclude that the present theme is in accordance with the patient narratives in the raw data. |
| This is my personal bias, but the term liver cirrhosis is redundant and just cirrhosis should be used. | We have followed your advice and changed to cirrhosis throughout the text. |
| The term “caregiver” is often used in the literature to refer to a family member who provides supplementary care. Healthcare provider or healthcare professional would be a more proper terms for this paper. | Thank you for helping us to clarify this. Accordingly, we have changed this and now use healthcare professional (HCP) in the manuscript. |
| *Methods:* |  |
| How were the 20 patients selected for individual interviews? | From the medical records, we obtained a list of eligible patients for the interview. In order to obtain a wide variety in the patient narratives, patients were consecutively asked in order to achieve a purposive varied sample, i.e. background characteristics, which is evident on page 5, lines 124-126 and table 1.  To simplify the text, we have removed text regarding 20 interviews and now focus on the 18 that were included in this analysis, page 5, line 126. |
| *Results:* |  |
| Informant CPT score or class should be included (in addition to the number of years with cirrhosis) | Information regarding CPT score may be reported. Since the CPT score is a dynamic measure, we are doubtful about what the information adds. We have chosen to clarify in the ‘Strengths and limitations’ section that “Several participants had previous experience with a higher Child Pugh class, i.e. B or C. The result thus reflects experiences from different stages of cirrhosis”. Please see page 21, lines 487-489. |
| *Discussion:* |  |
| The authors on a few occasions talk about stigma and reluctance to seeking care in cirrhosis – is this a finding of their study? If not and is more a reference to prior literature, I would advise the authors minimize this as their own findings show that patient did not feel stigmatized or show reluctance to seek care (likely due to sample bias in this case). | We have reduced the discussion regarding stigmatisation and now solely refer to previous literature; please see page 18, lines 402-404. Further, we have discussed the lack of experienced stigma in meetings with HCP in the strengths and limitations section. Page 21, lines 475-477, and lines 481-483. |
| Discussion really needs to be more succinct and focus on what a clinician should take a way to change practice while emphasizing the 2 themes they found. Additionally, the focus should be on authors think healthcare providers need to accomplish change in practice – education, training, e-tools, handouts. | Based on your suggestion, we have re-worked the whole discussion section, please see page 17, line 392 to page 19, line 444. Further, we have highlighted clinical implications on page 18, lines 406-408; page 19, lines 428-431 and lines 439-442. |
| As an example, line 353-354: “However, the findings disclose communication barriers that inhibit patients’ right to be involved in the conversation [23].” Is this a finding of their analysis or reference 23 | The sentence has been rephrased; please see page 18, lines 399-400. |
| Right now, the first 2 paragraphs of the discussion spend a lot of time reviewing the literature. It is hard to tell what is new from this study and what has already been shown. As such, the two paragraphs should be combined as both emphasize the need for “person-centered” communication and care and more streamlined. | Based on your suggestion, we have rephrased and combined the first two paragraphs; see page 17, line 392 to page 18, line 408. We have moved all motives regarding person-centred care to the last section of the discussion where we discuss person-centred care, see page 19, line 445 to page 20, line 468.  Further, we have strived to clarify the whole section regarding what statements come from our own data or previous research, respectively. Please see page 17, line 392 to page 19, line 444. |
| Lines 370-378 represent a more succinct and focused writing style where I walked away with a clear understanding of the results in the context of prior literature as well as what I should do differently. | Thank you for highlighting a good example. |
| Similarly, in the next paragraph - lines 362-367, what do the authors suggest as a change – longer visits, more educational material between visits, etc. | Our recommendations for the clinical implications, based on our findings and previous literature, have been added on the following pages; page 18, lines 406-408; page 19, lines 428-431 and lines 439-442. |
| The paragraph before limitations/strengths is also long, somewhat redundant and again ends with patient-centered care. But how does one achieve this? A review of literature to answer this question would be much more relevant to the discussion. | To reduce redundancy of person-centred care, we have concentrated all statements regarding person-centred care to the last paragraph in the discussion section, which has been thoroughly re-worked; see page 19, line 445 to page 20, line 468. Further, we have added a clinical implication on how to achieve person-centred care in practice; see page 20, lines 463-467. |
| If there isn’t much literature in this area, then what do that authors recommend given their interview with patients as aims for future studies? | Thank you for a good suggestion. We have developed the last sentence in the discussion section to clarify our thoughts on further studies; see page 20, lines 468-470. |
| Strength and limitations section does not list any real limitations. What biases were introduced based on how patients were enrolled and selected for the detailed intereview? | Thank you for this comment. We have completed the ‘strengths and limitations’ section with two sentences, according to your suggestions: “The purposive sampling may involve sampling bias, as patients feeling stigmatised or having negative healthcare experiences might have declined participation”. (Page 21, line 475-477) and “Hence, in future studies, it is important to ask specific questions regarding healthcare experiences”, which we did not do in this study. “Given this limitation …” (Page 21, lines 483-484) |
| Since patients were not specifically asked about cirrhosis related healthcare experiences, did this introduce any bias – missed themes that patients would have discussed if prompted in a more structured fashion? | Based on the comment above and also this comment, we have added information regarding the limitations during the interviews. “The fact that informants were not prompted to answer specific questions is a weakness that may prevent all perspectives, of the patients' care experience from being captured”. See page 21, lines 481-483. |
| How about response bias – of 168 patients enrolled, only 86 responses were used in the analysis? How about the fact this is just one center and one country - does it apply to other countries with other healthcare systems. | Thank you, we have now clarified that out of all 168 patients who answered the questionnaire, 86 chose to answer the two open-ended questions. Further, the patients represented all six hospitals involved in the study; see page 21, lines 485-487. Also, we have added information regarding transferability of our findings to other healthcare systems; see page 22, lines 508-510. |
| **REVIEWER 3** |  |
| Is the manuscript technically sound, and do the data support the conclusions? Reviewer #3: No | Thank you for your thorough review of our manuscript. We will respond to each of your questions and suggestions for improvements under each specified question below. |
| Has the statistical analysis been performed appropriately and rigorously?  Reviewer #3: N/A | N/A |
| Have the authors made all data underlying the findings in their manuscript fully available?  Reviewer #3: No | N/A since there are ethical considerations limiting our possibility to share data, which has been described previously. |
| Is the manuscript presented in an intelligible fashion and written in standard English?  Reviewer #3: No | The manuscript has been proofread by an experienced, native English speaker and professional language reviewer. We have thoroughly rewritten the introduction section and the discussion section according to each comment below. We hope that the new changes have improved the text and made it more intelligible. |
| First of all, I would like to congratulate you on the work you have done. I believe that learning about the experience of living with chronicity is a great objective and that the current literature lacks this type of study, which is so necessary in order to give patients a voice and thus increase the quality of their care. However, I believe that there are certain issues that need to be improved in order for this study to be published. | We appreciate that you see the benefit of our study and hope that our clarifications provide answers to the important questions you have raised. |
| *Summary* |  |
| In general it reflects the work done, however I think that if they included what type of caregivers they are referring to from the beginning it would better situate the reader. | Thank you for pointing this out. We have made the following clarification: “…experiences of *the physician-based* healthcare were also spontaneously uttered” page 4, lines 83-84. |
| Also, the location of where the study was conducted should be included in the material and methods. | We believe the context of the study has been sufficiently described on page 5, lines 126 to page 6, line 131 and in Figure 1. |
| Finally, the wording of your conclusions does not really correspond to the results of your study, so I would recommend that you rewrite this section taking into account your own data. | The conclusion sections in the abstract and in the main text have been revised, following your suggestion. Please see page 2, lines 46-53; and page 22, line 512 to page 23, line 525. |
| *Introduction* |  |
| In general, the whole introduction is very disorganised, jumping from one topic to another, without any thread of argument or order. I consider all the information they provide to be very relevant, as it situates the reader in the state of the question, but this disorder makes it difficult to read. | Thank you for your advice. We have thoroughly revised the introduction section to improve the readability. Also, we have moved the theoretical framework to the ‘Material and method’ section following your suggestion below. We hope the revised text is now easier to read. In the revision, we added two new references, Waibel 2018 and Britten 2020. Please see page 3, line 57 to page 4, line 98. Also, changes have been made in the introduction paragraph in the abstract, page 2, lines 26-31. |
| As in the summary, I would recommend that they talk about professional carers. | Based on another reviewer’s comment, we have now changed from caregiver to using healthcare professional (HCP) throughout the manuscript. |
| They also talk about some of the stigma attached to liver cirrhosis, but there is no depth and I think this is an important topic to delve into. | Thank you, we have added information about how stigma may be experienced in cirrhosis. Please see page 3, lines 70-71. |
| As for including Santana's conceptual framework in the introduction, I think it is totally incorrect. This framework should be explained in the methodology section and the introduction should be used to correctly situate the reader as to what is known and what remains to be known. | Based on your suggestion, we have moved the text regarding Santana’s conceptual framework to a new sub-heading ‘Theoretical framework’ after the heading ‘Analysis’ under the ‘Material and method section’. Page 9, line 202 to page 10, line 219. |
| In fact, I believe that this is something that is missing in your introduction and is a clear justification of the need to know the experience of those affected from their own perspective. | Also, we have rephrased the last paragraph in the introduction section to justify the rationale of our study; page 4, line 99 to page 5, line 109. |
| Furthermore, in their introduction they talk about the experience of chronicity in general, while later they focus their study on the patient-health professional relationship. Are there any studies that show that this relationship is not adequate? Why is there a need to listen to those affected themselves? | Based on your comment above, we have rephrased the whole introduction section, which now also states arguments on the importance of the patient-healthcare professional relationship. Please see page 3, lines 64-66, and page 4, lines 99-101. |
| I suggest first of all to put the ideas in this section in order and then to justify in a more concrete way the need for this study. | Based on your comments above, we have thoroughly reworked the introduction section. We hope the need for this study is made more concrete now. See page 3, line 57 to page 4, line 98. |
| *Material and Methods* |  |
| This section raises many doubts in my mind. They talk about an interpretative, descriptive and inductive design, but these are characteristics that are already presupposed in a qualitative research, therefore, I do not understand the need to expose it. | Thank you for your comment. We have followed Braun and Clarke’s thematic analysis and consider it important to be transparent to readers in our analytical decisions. Further, we used one theoretical framework and found it essential to describe how this was applied inductively, as presented at page 5, lines 112-115. |
| On the other hand, there is no mention at any point of qualitative theoretical foundations. Are we dealing with phenomenology, symbolic interactionism or do they intend to carry out a grounded theory study? I think that framing your study within one of the qualitative theoretical foundations would allow for a more robust design. | To clarify, we have added information in the ’Study design’ section that “Data were analysed using Braun and Clarke’s six step thematic ***analysis*** process…”. Please see page 5, lines 114-115. In the ‘Data analysis’ section, the theoretical foundations of this study are already apparent; see page 8, lines 171-173. |
| In terms of design it is confusing. First they do 18 semi-structured interviews and then they ask two open questions to 86 patients... What does this add? I think that with the 18 interviews there would be enough information to put together good results. So, this design is not correct and this makes the results inadequate. | Both interview transcripts and self-reported open-ended survey responses are equally considered as qualitative data. Further, we did not specifically ask participants about their healthcare experiences during the interviews. However, we did in the surveys. Consequently, to answer the study aim, all data, including interview transcripts and open-ended survey responses, were used. Therefore, we argue that the results are adequate, and answer the study aim, although data were collected with two different methods. |
| On the other hand, they talk about semi-structured interviews and in their script they do not include questions about health professionals? Or at least that is what they state in the data collection section. | In the data collection section and in the strengths and limitations section, we have strived to be transparent with data quality regarding spontaneously uttered interview data, as a natural part of the patients’ day-to-day life. Please see page 7, lines 156-160, and page 21, lines 478-483. We think that the transparency of this limitation is important for readers to evaluate the trustworthiness. |
| *Data analysis* |  |
| They do not reflect the work done, nor the training of the people who carried out the analysis | As further described below, we have provided details about the analysis process on page 8, lines 173-180. Based on your suggestion, we have added information regarding the author’s collaboration in the analysis process; please see page 8, lines 182-184. |
| Moreover, in my opinion, it is not necessary to explain Braun and Clarke's method so extensively and you should explain better how you adapted this method to your research. | The rephrased first section in the analysis paragraph, described on page 8, lines 174-180, refers to the analysis process performed, in accordance with Braun and Clarke 2006. |
| *Results* |  |
| It is impossible for me to know which of your results correspond to N=18 or N=86? Assuming that they are from the semi-structured interviews, | We consider all aspects of the patients’ healthcare experiences to be equally important, regardless if it has been expressed in interviews or open-ended survey responses. All data have thus been treated equally during the analysis and reporting. |
| I think that these results are not well presented, as certain statements are made that do not follow from the data, and therefore this should be in the discussion section. Perhaps it is for this reason that the results should be reduced, as they should be unified and not repeated throughout the whole section. | The study’s themes and sub-themes are, according to the qualitative research tradition, exemplified by raw rata codes. This was done to confirm that the results are grounded in research data. Three more quotations have been added to make the results even more robust.  Please see page 11, lines 246-248; page 13, line 277-282; and page 17, lines 376-381. |
| Furthermore, there are contradictions in her own results and this is not described later in the discussion. | Thank you for pointing this out. We have added two sentences regarding the contradictory findings; see page 20, lines 460-462. |
| There is an important part of the discussion that is not clear from the results. For example, they talk about stigmatisation and this has not appeared in their results | Following your suggestion, we have reduced the discussion section regarding stigmatisation and have now only referred to previous literature; please see page 18, lines 402-404. |
| They should rewrite it and emphasise the important issues. | Based on your suggestion, we have revised the discussion section in order to emphasise our findings; please see page 17, line 392 to page 19, line 444. |
| It would also be advisable to work more on the lack of training of health professionals in communication skills and to justify this lack of training with other studies that could confirm the results they have obtained. | We have added our suggestions for clinical implications from our own findings and previous literature; please see page 18, lines 406-408; page 19, lines 428-431 and lines 439-442. |
| In general, I consider that the objective is appropriate and the topic very interesting, but there are methodological errors that need to be improved to make the study more robust. | Thank you for your efforts in reviewing our manuscript. We have done our best to respond to each of your questions and made changes in the manuscript accordingly. We hope you will consider the changes appropriate, and sufficient. |
